# Botulinum Toxin Treatment for Limb Spasticity in Childhood Cerebral Palsy

**DOI:** 10.3389/fphar.2016.00029

**Published:** 2016-02-19

**Authors:** Vito Pavone, Gianluca Testa, Domenico A. Restivo, Luca Cannavò, Giuseppe Condorelli, Nicola M. Portinaro, Giuseppe Sessa

**Affiliations:** ^1^Dipartimento di Chirurgia Generale e Specialità Medico-chirurgiche, Sez. Ortopedia, Azienda Ospedaliera Universitaria Policlinico-Vittorio EmanueleCatania, Italy; ^2^Neurologic Unit, Department of Internal Medicine, Nuovo “Garibaldi” HospitalCatania, Italy; ^3^Humanitas Clinical and Research Center, Clinica Ortopedica e TraumatologicaMilan, Italy

**Keywords:** botulinum toxin A, cerebral palsy, multi-level treatment, key-muscle, resistance

## Abstract

CP is the most common cause of chronic disability in childhood occurring in 2–2.5/1000 births. It is a severe disorder and a significant number of patients present cognitive delay and difficulty in walking. The use of botulinum toxin (BTX) has become a popular treatment for CP especially for spastic and dystonic muscles while avoiding deformity and pain. Moreover, the combination of physiotherapy, casting, orthotics and injection of BTX may delay or decrease the need for surgical intervention while reserving single-event, multi-level surgery for fixed musculotendinous contractures and bony deformities in older children. This report highlights the utility of BTX in the treatment of cerebral palsy in children. We include techniques for administration, side effects, and possible resistance as well as specific use in the upper and lower limbs muscles.

## Introduction

Cerebral palsy (CP) is a group of permanent, non-progressive disorders of movement and posture resulting in activity limitations and forced dysmorphisms. CP is not a disease or a single entity but rather a complex cerebral disorder with final expression in different etiological events (Pavone and Testa, 2015). The etiologic events act in an early phase of fetal and infant cerebral development. The disorder not only affects motor activity but is also often associated with other disturbances involving sensation, perception, communication and behavior. Cognitive delay and epileptic crises are frequently observed in these patients.

CP is the most common cause of chronic disability in childhood occurring in 2–2.5%_0_ of births.

The risk of CP is 20–30 times more common in newborns of low birth weight. It is 1.4%_0_ for newborns 2500 g and 78%_0_ in newborns below 1000 g. Predisposing factors include maternal disorders such as gestosis, twin birth, intellective delay, miscarriages, placental anomalies as well as prematurity, low intra-uterine development, and neonatal encephalopathies (Pavone and Pavone, 2006).

Etiological factors that cause CP are primarily hypoxic ischemic encephalopathy and environmental conditions, but infections as well as metabolic, malformative, and genetic disorders are also frequently reported. Such conditions affect the CNS during pregnancy, at birth, or during the first 2 years of postnatal life and act on the first motor neuron and decrease the excitatory and regulatory input. The lesions from the cortex through the reticulo-spinal and cortico-spinal tracts reach the lower motor neuron localized in brain stem and spinal cord (MacLennan, 1999; Moreno-De Luca et al., 2012). The anomalous input results in impaired motor control and increased alfa-motoneuron excitability causing spasticity.

Several CP classifications have been proposed based on the localization of the cerebral lesions and their clinical expression. In the past, the spastic form was subdivided into hemiplegic, diplegic, and tetraplegic; the dyskinetics include choreoathetotic, dystonic, ballism, ataxic, and finally a mixed form (Cowan et al., 2003; Morris, 2007). More recently, CP classification has been based on: (1) the type and severity of motor abnormalities; (2) the anatomical distribution of the extremities; (3) the characteristics of the neurologically associated dysfunction; and (4) on the timing of the presumed causal event (Surveillance of Cerebral Palsy in Europe, 2002; Christine et al., 2007; Rosenbaum et al., 2007; Pavone and Testa, 2015).

The “spasticity” sub-type is the most frequent form and is defined as muscular resistance to stretching or as an excessive or involuntary muscle activity. Spasticity is characterized by an initial resistance to passive movement, followed by a sudden release called the clasp-knife phenomenon. It is associated with brisk tendon reflexes and an extensor plantar reflex, clonus, diminished active movements, and disuse atrophy (Haslam, 2007). Spasticity may result in contractures, pain, and bone lesions with easy fractures. On the other hand, contractures may worsen or cause spasticity (Ward, 2012).

To measure motor abilities and functional limitations, many scales and classification systems have been proposed. One of the most common is the Gross Motor Function Classification System-Expanded and Revised (GMFCS-ER) proposed by Palisano (Palisano et al., 1997, 2008; Rethlefsen et al., 2010). This test distinguishes the functional anomalies according to a scale with five levels. In level I the patient walks without limitations; level II walks with limitations; level III walks using a hand-held mobility device; level IV has limited self-mobility and may use powered mobility; and level V may be transported in a manual wheel chair.

Other systems include the Functional Mobility Scale (FMS; Graham et al., 2004) that is based on interviews with the parents. This describes the assistance needed for the child to cover three distances (5, 50, and 500 yards or meters). To assess manual functionality, the Bimanual Fine Motor Function (BFMF; Beckung and Hagberg, 2002) and Manual Ability Classification System (MACS; Eliasson et al., 2006) and for Communication Function (CFCS; Hidecker et al., 2011) have been proposed. Other systems have been applied to gait (classification of gait analysis), dystonia [Barry-Albright Dystonia Scale (BAD)], Dystonia Impairment Scale (DIS; Monbaliu et al., 2012), and Burke Scale (Burke et al., 1985), as well as for drooling, pain, speech, and oromotor dysfunction (Pavone and Testa, 2015).

The treatment of CP uses different elements including communication, social development, emotivity, education, daily activity, physical aspect, nutrition, and mobility. Physical therapy goals include stretching, physiotherapy, hydrotherapy, elective stimulations, and hot/cold treatments.

Pharmacologic therapy includes systemic and focal treatments. Systemic anti-spasticity drugs include baclofen and diazepam (used only for short duration). Systemic drugs can be effective in diffuse spasticity and affect several muscular groups. However, they are often associated with a number of intolerable systemic side effects including dizziness, sedation, confusion, nausea, and vomiting, fatigue, lower seizure threshold, and central nervous system depression. Focal treatments are mainly represented by botulinum toxin (BTX). The advantage of focal BTX is that it directly treats the symptomatic muscles. When used at recommended doses, it avoids systemic side effects. This elimination of spastic components is mandatory in CP treatment because it allows affected individuals to use the residual component of selective motor control more effectively and functionally (Rosenbaum et al., 2002; Majnemer and Mazer, 2004).

The use of BTX has become popular for orthopedics as a treatment for spastic and dystonic muscle. The success of BTX depends on the adjunctive use of physiotherapy and/or splinting or casting (Ramachandran and Eastwood, 2006). Moreover, BTX treatment has demonstrated a beneficial effect on the reduction of pain associated with focal muscular hyperactivity (Wissel et al., 2000). In fact, in spasticity, the spasmodic muscle contraction induces extreme vasculature compression because of the simultaneous activation of adjacent motor units. Ischemia eventually gives rise to nociceptive pain (ischemic muscular pain). As soon as the spasm ends, the blood flow returns to normal, and the pain quickly recedes. BTX improves muscular pain and reduces this vicious circle by inducing muscle relaxation (Restivo et al., 2003).

## The neuromuscular junction

The neuromuscular junction (NMJ) is a synaptic interface between a branch of the motor neuron and muscle fibers. It is composed of three elements: pre-synaptic (motor nerve terminal), intrasynaptic (synaptic basal lamina), and post-synaptic (muscle fiber and muscle membrane; Cowan et al., 2003).

## Botulinum toxin. mechanism of action

Clostridium botulinum produces non-toxic proteins and a complex mixture of proteins containing botulinum neurotoxin (Surveillance of Cerebral Palsy in Europe, 2002). There are seven serotypes of neurotoxin: Botulinum toxin A (BTX-A), Botulinum toxin B (BTX-B), Botulinum toxin C (BTX-C), Botulinum toxin D (BTX-D), Botulinum toxin E (BTX-E), Botulinum toxin F (BTX-F), and Botulinum toxin G (BTX-G). All of these proteins can inhibit release of acetylcholine from nerve terminals, but there are some differences about their potency, duration of effect and intracellular protein targets (Jankovic and Schwartz, 1995; Aoki and Guyer, 2001).

This mechanism is unique to the mechanism of action of tetanus toxin. For this reason, BTX has been used in focally contrasting the muscular hyperactivity associated with tetanus infection as well as in the treatment of muscular hyperactivity not-associated with spasticity (Restivo et al., 2002; Restivo and Marchese-Ragona, 2006; Verderio et al., 2006). BTX-A reduces muscular activity in a dose-dependent manner. The other BTX serotypes cleave different proteins of the complex (Arnon et al., 2001; Koman et al., 2004).

In about 4 weeks, turnover of the SNARE protein complex allows exocytosis of acetylcholine to resume. Nerve conduction is re-established by new axonal sprouting and elongation of the endplate and by retraction of the new axonal sprouts (de Paiva et al., 1999). Clinically, muscle relaxation lasts for 12–16 weeks.

## Techniques for administration

The principal commercial preparations of botulinum toxin are: Botox [OnabotulinumtoxinA, 100 International Units (IU) per vial; Allergan Inc, Irvine, California]; Dysport (AbobotulinumtoxinA, 500 IU per vial; IpsenLdt, Slough, United Kingdom) and Xeomin, a more recently introduced neurotoxin free of complexing proteins (IncobotulinumtoxinA, 100 IU per vial; MerzPharma, Germany). Several studies have addressed the topics of equivalency ratio among the different BTX-A preparations. Comparative human experimental studies support dose conversion ratios between Dysport and Botox (ratio D/B) less than 3:1 (Wohlfarth et al., 2009). The conversion ratio between Botox and Xeomin is reported to be 1:1 (Poulain et al., 2013).

To maximize the clinical effectiveness of BTX-A, the toxin must be injected inside the fascia compartment of the muscle in a dose suitable of neutralizing the neuromuscular junction activity and in an adequate volume such that diffusion to the junctions at the end-plate area occurs while unwanted spread is minimized. The most effective dosage for the affected muscle is not clear, but it changes with density of neuromuscular junctions in any given muscle (Alhusaini et al., 2011). The indicative recommended doses are 12 units/kg for Botox (Francisco, 2004) and 30 units/Kg for Dysport (Graham et al., 2000).

Age since 2 years is the best for treatment of lower extremities for regional or focal impairment and 2 years too for the upper extremities. There are several muscles involved. The neuromuscular junctions have age-dependent differences. They are smaller and higher density in young patients than in adults (Ma et al., 2002). The dosage must be correlated with weight, size/volume, level of hypertonia, number of muscles to be injected, and the underlying weakness. The injection of BTX could be administered under local or general anesthesia or with conscious sedation. This depends on patient age, muscular districts, the number of sites to be treated, and underlying pathology (Koman et al., 2003).

The timing of the injections is controversial. Most clinicians agree that the earlier the spasticity is reduced, the better the outcome. Botulinum toxin can be injected as early as 18 months of age. There is no upper age limit, however. Once the muscle is shortened with advanced age, the effect of spasticity relief will not be as apparent because of contracture (Koman et al., 2003). The dose advice is for a total maximum dose of 15 IU/kg or a maximum of 400 IU per injection site. This is a maximum of 50 IU and a maximum volume per site of 0.5 ml. Longer muscles may require two or more injections. To better focalize the target muscles, BTX injection is usually performed under electromyographic (EMG) or ultrasound guidance (Chin et al., 2005).

## Botulinum toxin: Side-effects

Side-effects are rare when the dosing and technique protocols are correctly followed (Koman et al., 2003). Adverse events can be located into the site of injection or can be distant from the site of injection. Pain at the site of the injection is unlikely a clinical problem and is usually moderate. The most common side-effect is weakness in adjacent muscles caused by diffusion of BTX across the muscle boundaries (Graham et al., 2000). The BTX diffusion is strictly dependent on the dilutions that are being used. The larger the dilution, the higher the risk of diffusion into adjacent muscles. However, a higher dilution can also represent a strategic choice of physician. This is especially true for bigger muscles in which a larger diffusion of BTX may induce a more remarkable improvement in spasticity. Side-effects distant from the site of injection are unusual and consist of generalized weakness, flu-like syndrome, urinary incontinence, constipation and dysphagia. The most serious side-effect is aspiration pneumonia. This is critical in CP patients with general involvement and pre-existing pseudobulbar palsy because in these patients, small amounts of BTX can worse pharyngeal function (Koman et al., 2003).

Localization of muscles to be injected is based on palpation. When using BTX, it is important to distinguish large from small muscles. In fact, large muscles, typically in lower limbs, could be manually individuated with an accuracy rate of 46–78% (Jankovic and Schwartz, 1995). This is different from small muscles. Other methods used to localize the target muscles include ultrasound, electromyography, and electrical stimulation.

## Resistance

A small percent of children may not respond to initial injection of botulinum toxin. It is important to consider contraindications before classifying the patient as a “non-responder.” A non-responder is a child who shows a relative or complete loss of effect after a second injection. Factors contributing to an inadequate clinical response include inadequate dosage, inappropriate goals, or excessive weakness in the affected muscles. Neutralizing antibodies have also been reported to cause an inadequate response (Chin et al., 2005).

Resistance to botulinum toxin therapy is characterized by absence of any beneficial effect and by lack of muscle response following injection. Antitoxin antibodies are presumed to be responsible for most cases of resistance. Using the smallest possible effective dose can prevent development of an antitoxin antibody. Providers can also extend the time interval between treatments to at least 3 months. The use of Botulinum toxin B or F may be beneficial to patients who have developed antibody resistance (Love et al., 2001).

## Indications for use in cerebral palsy

As the child develops, the spastic muscles frequently fail to grow as rapidly as neighboring structures. This causes contractures, deformity and consequent impairment of function. The muscular hypertonia may be the primary cause of shortening because even normal muscles will become shortened if the tone is persistently increased (Huet De La Tour et al., 1979). A similar failure of growth occurs in animals with increased muscle tone such as the hereditary spastic mouse (Ziv et al., 1984). In contrast, passive stretching of relaxed muscles may restore normal longitudinal growth (Holly et al., 1980). Based on this premise, it has been postulated that this effect could be harnessed to relax overactive spastic leg muscles by BTX A injections. This hypothesis has been tested in animal models. BTX A restored the muscle length to normal and significantly shortened the Achilles tendon length in the hereditary spastic mice. It did not induce changes in normal mice (Cosgrove and Graham, 1994).

Injections of BTX determine weakening in spastic muscles encourages muscle strength and hence muscle growth while allowing the weak antagonists to be strengthened. This mechanism avoids the development of bony deformities secondary to abnormal muscle pull and contracted tendons and joints. Moreover, a combination of physiotherapy, casting, orthotics and injection of BTX may delay or decrease the need for surgical intervention reserving single-event multi-level surgery for fixed musculotendinous contractures and bony deformities, which are common in older children. The success of botulinum toxin administration depends on many factors. Patient selection is critical. Children with spasticity who do not have fixed contractures benefit a great deal from treatment, but patients with dyskinesia have a variable response. Patients with athetoid signs do not benefit at all (Gough et al., 2005; Lannin et al., 2006).

Treatment goals are to attain the next motor milestone, to allow patient verticalization and to achieve the best possible locomotion. If the child can walk, the intention is to maintain, improve, and optimize mobility. In the case of stagnation on a lower motor level, the aim is to maintain, improve, and optimize motor function at this level (Koman et al., 2003).

### Upper limb muscles

In selecting target muscles for BTX injection, it is useful to evaluate functional deficits and postural deformity and to analyze the relationships between muscular hypertonia and individual abilities and disabilities (Lannin et al., 2006). The most common muscles injected are the biceps brachii for elbow flexor spasticity, pronator teres for pronated forearm, flexor carpi radialis and flexor carpi ulnaris for wrist flexion spasticity, and adductor pollicis for the thumb. The triceps brachii, pectoralis muscles, teres major, and deltoid have been injected for shoulder deformity (Preiss et al., 2003). There are several injection techniques for upper limb muscles. To identify the largest muscles, anatomic knowledge and palpation are sufficient. One should find the largest bulk of the muscle and inject the toxin mid-belly. The BTX dose guidelines for upper limb muscle spasticity are reported in Table 1.

**Table 1:** **General guidelines for use of BOTOX for upper extremity spasticity (Preiss et al., 2003)**.

**Muscles injected**	**Dose range (IU/Kg of bw)**	**Number of sites**
Biceps	2	2–3
Pronator teres	1	1
Flexor carpi radialis	2	1
Flexor carpi ulnaris	2	1
Flexor digitorumsuperficialis	2	1–2
Flexor digitorumprofundus	2	1–2
Flexor pollicislongus	0, 5–1	1
Adductor pollicis	0, 5–1	1

### Lower limbs muscles

Several studies have shown the short-term efficacy of BTX-A in the management of spasticity in lower limbs muscles with improvements in deformity and gait. Injection into the gastrosoleus is typical for treatment of spastic hemiplegic or diplegic children including dynamic gait and complex gait patterns relate to the equines. It this last case, the equinovarus deformity could be corrected with injection on the area of tibialis posterior or tibialis anterior. Injection in the hamstring is used to treat dynamic flexed genu caused by over-activity of these muscles. The good response to injection is due to hamstring lengthening or adductor tenotomy. Injection of the hip flexors is more technically demanding and may require ultrasound guidance for accuracy (Graham et al., 2000; Preiss et al., 2003; Moelenaers et al., 2013).

Muscles to be injected within the adductor or hamstring group and the appropriate techniques are under debate. In particular, children with wide involvement may have improvements in position. Patients with progressive subluxation of the hip may have improved pain and stiffness after injection of the involved adductors and iliopsoas, especially when combined with bracing of the hip in abduction. A reduction or stabilization of the hip migration percentage may occur—especially in children under the age of 2 years whose initial subluxation shows a migration percentage >30%. The BTX dose guidelines for lower limb muscle spasticity are reported in Table 2.

**Table 2:** **General guidelines for use of BOTOX for lower extremity spasticity (Preiss et al., 2003)**.

**Muscles injected**	**Dose range (IU/Kg of bw)**	**Number of sites**
Iliopsoas	2	2
Quadriceps	3–6	4
Medial hamstrings	3–6	3–4
Lateral hamstrings	2–3	2
Adductors	3–6	2
Gastrocnemius	3–6	1–2
Soleus	2–3	1
Tibialis posterior	1–3	1

## Post-injection treatment

The antispastic effect appears within 24 h to 3 days after injection and becomes maximum at 10 days to a month. It lasts for 3–6 months. Some patients are golden responders in whom the antispastic effect lasts for over a year. Proper exercises, splinting and casting may increase the number of golden responders. Casting for 2–3 weeks after injections may improve the results, (Pidcock et al., 2005), although it use as a method of applying stretch at the time of the injection is inappropriate since the reduction in stretch reflex and spasticity has not yet had the time to completely manifest.

## Conclusion

Botulinum toxin is a powerful neurotoxin that has an established place in the treatment of spasticity in CP. It can be used as early as 2 years of age and can be combined with other treatment options as the child grows older when untreated spasticity begins to cause contractures and deformities. The most common indications are young diplegic and hemiplegic children. The complex gait patterns often require treatment at multiple levels in a manner similar to the development of single-event multilevel surgery (Heinen et al., 2010).

Factors limiting its use are the high cost that implies a restriction on the frequency of treatment and the use of a maximum dose per each session. Moreover, before selecting the target muscles, it is important that a detailed clinical analysis be performed including differentiation of dynamic and structural components of deformity as well as definition of a treatment goal for the specific patient.

## Author contributions

All authors listed, have made substantial, direct and intellectual contribution to the work, and approved it for publication. In particular, VP, GT, LC and GC treated the orthopedic aspect, DR and NP treated pharmacological topic, GS revised the manuscript.

### Conflict of interest statement

The authors declare that the research was conducted in the absence of any commercial or financial relationships that could be construed as a potential conflict of interest.
